# Systemic oncological treatments in patients with advanced pancreatic cancer: a scoping review and evidence map

**DOI:** 10.1007/s00520-022-07564-8

**Published:** 2023-01-09

**Authors:** Josefina Salazar, Javier Bracchiglione, Roberto Acosta-Dighero, Nicolas Meza, Adriana-G. Meade, María Jesús Quintana, Carolina Requeijo, Gerardo Rodríguez-Grijalva, Marilina Santero, Anna Selva, Ivan Solà, Xavier Bonfill, Appropriateness of Systemic Oncological Treatments for Advanced Cancer (ASTAC) Research Group, Karla Salas, Alba Antequera, Ariadna Auladell-Rispau, Gerard Urrútia, Pamela Meinardi, Juan Irassar, Daniel Simancas, Rosario Dorantes, Angela Merchán, Yahveth Cantero, Edgar Hernandez, Miguel Sugrañes

**Affiliations:** 1 Iberoamerican Cochrane Centre, Biomedical Research Institute Sant Pau (IIB Sant Pau), Barcelona, Spain; 2 Interdisciplinary Centre for Health Studies (CIESAL), Universidad de Valparaíso, Viña del Mar, Chile; 3 CIBER Epidemiología Y Salud Pública (CIBERESP), Barcelona, Spain; 4 Universitat Autònoma Barcelona, Barcelona, Spain; 5 Corporació Sanitària Parc Taulí, Sabadell, Spain

**Keywords:** Pancreatic neoplasms, Antineoplastic agents, Immunotherapy, Review literature as topic

## Abstract

**Purpose:**

To identify, describe, and organise currently available evidence regarding systemic oncological treatments (SOTs) (chemotherapy, targeted/biological therapies, and immunotherapy) compared to best supportive care (BSC) for patients with advanced pancreatic cancer (PC).

**Methods:**

We conducted a scoping review and evidence mapping, adhering to PRISMA-ScR checklist. We searched MEDLINE, EMBASE, Cochrane Library, Epistemonikos, PROSPERO, and clinicaltrials.gov for eligible studies. We included systematic reviews (SRs), randomised controlled trials (RCTs), quasi-experimental, and observational studies evaluating SOTs compared to BSC or no treatment in patients with advanced PC. Two independent reviewers performed the screening process and data extraction. We developed evidence maps as an interactive visualization display, including the assessed interventions and outcomes.

**Results:**

Of the 50,601 records obtained from our search, we included 43 studies: 2 SRs, 16 RCTs, 4 quasi-experimental studies, 20 observational studies, and 1 protocol for a quasi-experimental study. Forty-two studies reported survival-related outcomes and most favoured SOTs, while five reported toxicity and most favoured BSC. Other patient-centred outcomes, such as quality of life, were scarcely reported.

**Conclusions:**

This study highlights the current evidence gaps in studies assessing treatments for patients with advanced PC, mainly the lack of reports of non-survival-related outcomes, pointing out research areas that need further attention to make better recommendations for these patients.

**Supplementary Information:**

The online version contains supplementary material available at 10.1007/s00520-022-07564-8.

## Introduction

Pancreatic cancer (PC) is an important public health problem, as it has the highest incidence-to-mortality ratio of any solid tumour, and it represents 2.6% of total new cancer cases. It accounted for almost as many deaths as new cases in 2020 and represented the seventh leading cause of cancer death in both sexes worldwide [1].

Survival rates are significantly lower when diagnosed at an advanced stage, having a 5-year survival rate of 14.4% and 3% for regional and distant stages at diagnosis, respectively [2]. Therefore, patients with advanced PC have a high risk of dying in the short or medium term, which is conceptualised as the ‘end of life’ (EoL) period [3]. Deciding the most appropriate treatment for patients with advanced PC at the EoL should be based on the best available evidence and considering patient’s values and preferences, since failing to do so could increase patients’ psychological distress and the overutilisation of treatments that may be inconsistent with personal preferences [4, 5].

Therapies prescribed for PC are systemic oncological treatments (SOTs) such as chemotherapy, targeted/biological therapies, and immunotherapy. These treatments continue to be the preferred therapeutic approach for patients with advanced-stage PC since current clinical practice guidelines (CPGs) associate them with an improvement in survival outcomes [6–8]. Nevertheless, their usage is also associated with important side effects and toxicity [9].

On the other hand, a conservative strategy based on providing only the best supportive care (BSC) might constitute a valid therapeutic option. BSC constitutes a comprehensive approach focused on symptoms control and improvement of patients’ quality of life, usually involving multidisciplinary teams [10, 11]. Therefore, it represents a therapeutic option with lower toxicity, which is highly valued by patients [12, 13].

It is crucial to note that available CPGs’ recommendations are primarily based on the results of few experimental studies comparing SOTs with each other rather than with BSC, and on their modest survival-related outcomes differences. In contrast, our recent overview of systematic reviews (SRs) based on randomised clinical trials (RCTs) revealed contradictory results and a high uncertainty over the benefits of SOTs on overall survival when compared to BSC [14]. Furthermore, even though treatment decisions could profoundly affect patients’ quality of life, other important outcomes were rarely reported, such as toxicity, functional status, hospital admissions, symptoms, and quality of death [14].

Our overview presented an assessment of the evidence from SRs and, although comprehensive, included few SRs with a limited number of RCTs. Hence, it is key to complement these findings with other available primary studies, and thus get a clearer picture of the whole body of evidence regarding SOTs versus BSC for patients with advanced PC.

Scoping reviews and evidence maps provide a visual approach on the studies that have been conducted leading to the identification of areas that need further research (evidence gaps). Therefore, we developed a scoping review and evidence map to identify, describe, and organise the currently available evidence about the efficacy of SOTs (chemotherapy, biological/targeted therapies, and immunotherapy) compared to BSC for patients with advanced PC, considering all important patient-centred outcomes.

## Methods

We conducted a comprehensive scoping review and evidence mapping [15], adhering to the Preferred Reporting Items for Systematic reviews and Meta-Analyses extension for Scoping Reviews (PRISMA-ScR) checklist [16]. The protocol for this study was prospectively registered and is publicly available in Open Science Framework [17].

### Eligibility criteria

We used the PICOT framework (Patients, Intervention, Comparison, Outcomes, Type of study) to guide our eligibility criteria [18].

#### Type of patients

We considered eligible studies including adult patients (over 18 years of age) with PC, primary or recurrent, in stage III or IV, or described as advanced or metastatic by the study authors at the moment of the intervention, as these stages represent the EoL period. We excluded pancreatic neuroendocrine cancers.

#### Type of interventions

For the intervention arm, we considered any SOT (chemotherapy, biological/targeted therapy, or immunotherapy), either individual or combined, with or without supportive care. We excluded studies that considered only surgery or radiotherapy as intervention, as well as studies that considered only chemotherapy as adjuvant or neoadjuvant therapy.

We considered as comparator any supportive treatment, administered with the purpose of symptomatic or palliative control. This includes either usual treatment or BSC [11]. Studies that did not explicitly define the intervention of the control group, or studies with placebo as control group, were also included. We excluded studies if the control group considered any type of SOT (chemotherapy, biological/targeted therapy, or immunotherapy). We also excluded comparisons comprehending an intervention with non-palliative intent, such as surgery or radiotherapy with curative intent.

#### Type of outcomes

We considered the following outcomes:

- Overall survival: As a dichotomous outcome (at 3, 6, 9, 12, 24 months) and as a continuous or time to-event outcome.
- Progression-free survival: As a dichotomous outcome (at 3, 6, 9, 12, 24 months) and as a continuous or time-to-event outcome.
- Quality of life: Measured with validated scales.
- Functional status: Measured with Karnofsky or ECOG scale.
- Toxicity: Measured as moderate or severe adverse events, according to standardised classification.
- Symptoms related to the disease: Measured with validated scales that assess one or more symptoms.
- Admissions to hospital or long-term centre, or emergency consultations: Measured as the total number of admissions and days of admission during the follow-up period.
- Quality of death:
  - Admission to hospital at the end-of-life: Admission to the hospital in the last 30 days of life.
  - Palliative care provided during the last year: As a dichotomous outcome.
  - Place of death: Home, institutionalised (health community centre or residence), hospitalised (intensive care or other).

#### Type of studies

We included SRs, RCTs, quasi-experimental studies, and observational studies assessing the impact of SOTs in advanced or metastatic PC. In the case of SRs, we considered only those published from 2008 onwards. We did not apply any language restrictions or publication date restrictions to primary studies.

We considered as a SR any type of secondary research that states: i) an explicit research question, ii) a structured search strategy (defined as explicit search terms and data frame, in at least two databases), iii) explicit inclusion criteria and screening methods, iv) an explicit assessment of the quality or risk of bias of each included study, and v) an explicit approach to data analysis and synthesis [18, 19]. We considered as a RCT any experimental primary study with a randomised allocation of interventions. We considered as quasi-experimental studies those with an inadequate randomisation process, or specific study designs with a non-random allocation of interventions, such as interrupted time series or before-after studies. We considered as observational studies all the case-control and cohort studies, as long as they were controlled and had, at least, 30 included patients.

We excluded descriptive studies, CPGs, case reports, and non-SRs (such as narrative reviews).

### Search methods for identification of studies

We performed electronic searches in MEDLINE (access via PubMed), EMBASE (access via OVID), the Cochrane Database of Systematic Reviews, CENTRAL and Epistemonikos from inception until December 2, 2019. We designed search strings adapted to the requirements of each database that combined controlled vocabulary and search terms related to the main concepts of our clinical question. Since this study is part of a wider project, the search strategy included terms for pancreatic, hepatobiliary, and gastroesophageal cancer [17]. The search strategy for PubMed can be found in the online resource 1.

We also searched in PROSPERO and clinicaltrials.gov to identify protocols of potentially eligible studies. We asked experts in the field for relevant studies. We did not use any other strategy to search for grey literature.

### Selection of studies

Two reviewers independently screened titles and abstracts of the retrieved search results. A third reviewer solved disagreements. Afterwards, two reviewers independently conducted the full-text screening, with a third author solving any disagreement. For all this process we used Covidence (www.covidence.org).

### Data extraction

Two reviewers independently extracted data from the included studies, using a previously piloted data extraction sheet (Google Forms). For each included study, we extracted the following data: year of publication, country, study design, total number of studies included regarding our question (for SRs), total number of patients included (for primary studies), interventions (chemotherapy, biological/targeted therapy and/or immunotherapy), comparators (BSC, placebo, or non-specified), outcomes reported, direction of effect, defined according to its statistical significance as ‘favours intervention’, ‘favours comparison’, or ‘no differences’, and conflicts of interest.

### Data synthesis and analysis

We described the results of our search in a tabular view, classifying each included study by type of intervention assessed, methodological design, reported outcomes and direction of the effect. We used evimappr [20], an R package, to produce the bubble plots for the evidence map. We presented an interactive visualisation display that includes the interventions (chemotherapy, biological/targeted therapy, immunotherapy) in rows, and the outcomes in columns. The grids were populated with the corresponding studies in each intersection, classified by study design (SR, RCT, quasi-experimental study or observational study). Due to space limits, if a column (i.e. outcome) did not contain any study for any intervention, this was not plotted within the interactive bubble plot. Thus, we showed a more detailed scheme in a complementary static figure where we identified evidence gaps as those spaces on the grid that did not contain studies.

## Results

### Study selection

**Fig. 1:**
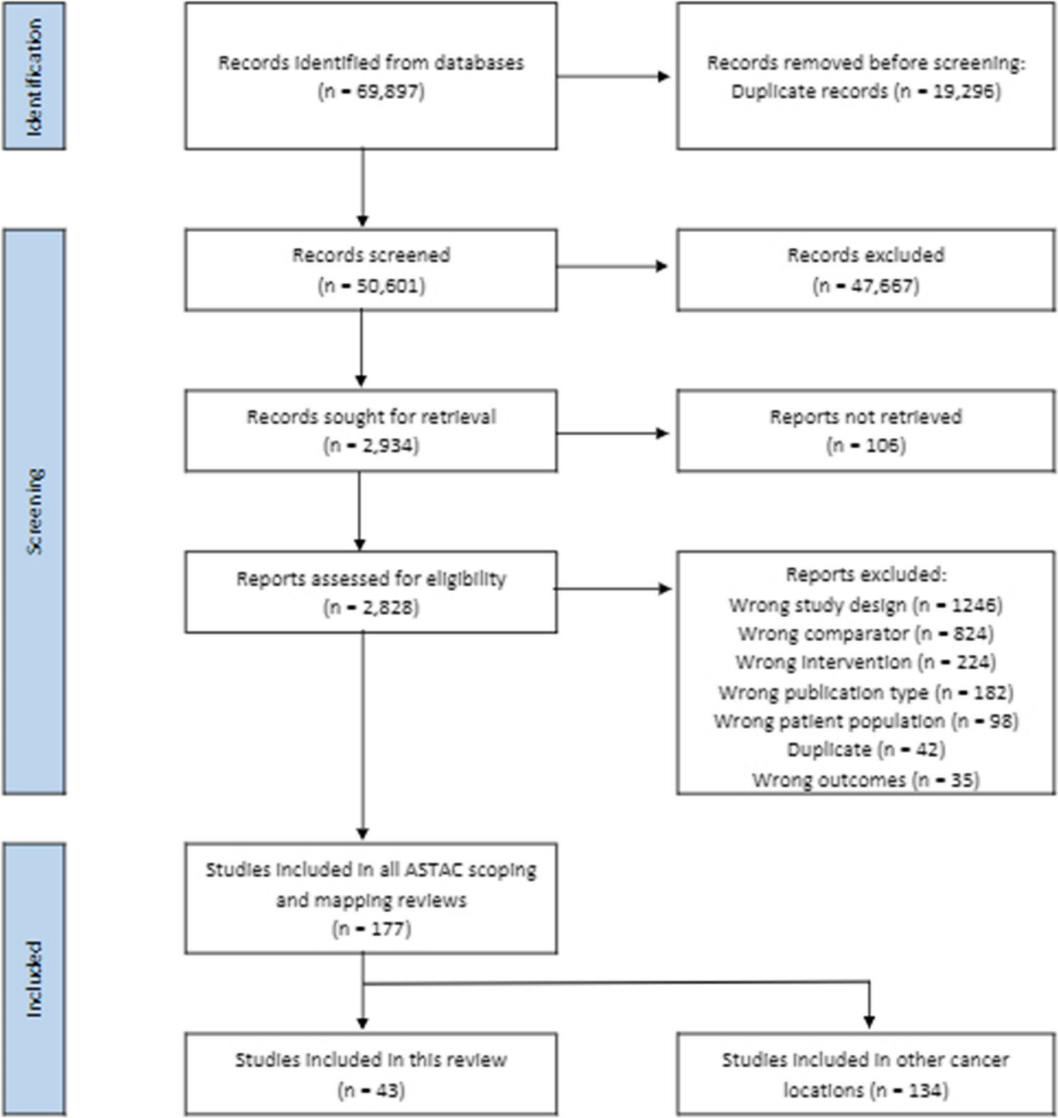
PRISMA flowchart

Our search returned 50,601 records for all cancer locations (hepatobiliary, gastroesophageal and pancreatic) once duplicates were removed. After title and abstract screening, we excluded 47,667 references. Of the 2,934 references, we were not able to retrieve 106 reports. Therefore, we reviewed the full text of 2,828 articles. We included a total of 177 studies for all cancer locations, of which 43 included participants with PC. Of these, 2 were SRs, 16 RCTs, 4 quasi-experimental studies, 20 were observational studies, and 1 was a protocol for a quasi-experimental study. Figure 1 summarises the screening process.

### Characteristics of the included studies

1. Chemotherapy
  36 studies published from 1979 to 2018, compared chemotherapy to either BSC (*n* = 24), or to an intervention not clearly specified (*n* = 12) for advanced PC. Among these, 34 were primary studies (11 RCTs, 3 quasi-experimental, and 20 observational) and included a number of participants that ranged from 31 to 303 for RCTs, 47 to 90 for quasi-experimental and 39 to 1085 for observational studies. We included two SRs with a total number of primary studies of 50 and 60, while the number of primary studies included in those SRs relevant to our question were six and nine (Table 1).
  The interventions assessed in primary studies included both monotherapy and combination therapy. Of those evaluating monotherapy, seven used gemcitabine, three fluorouracil, two S-1 and one glufosfamide. Combination therapies were 5-FU based (*n* = 9), gemcitabine–based (*n* = 3) and both 5-FU and gemcitabine-based (*n* = 5). Four studies did not specify the type of chemotherapeutic agent used. Both SRs assessed combination therapy. Most studies (64%) did not specify the line of therapy. Fifteen primary studies reported co-interventions, namely stents (*n* = 2), endoscopic procedures (*n* = 2), palliative surgery (*n* = 3), and radiotherapy (*n* = 8). One SR included radiotherapy and palliative surgery as co-interventions (Table 1).
  In addition to the 36 studies mentioned, we found one protocol for a quasi-experimental study that will compare gemcitabine-based chemotherapy to BSC (Table 1).
  Twenty-two (59.45%) of the studies did not report conflicts of interest. Of the 15 (40.54%) that did, 12 stated that there were no conflicts of interest to disclose (Table 1).
2. Immunotherapy
  Four studies published from 2010 to 2017, compared immunotherapy to either BSC (*n* = 2) or placebo (*n* = 2) for advanced PC. Among these, two were RCTs and two were quasi-experimental studies and included a number of participants that ranged from 47 to 154. All the studies assessed different immunotherapy agents, two as first line therapy and two did not specify the therapy line (Table 1).
  All studies reported their conflicts of interest, of which three (75%) stated that there were no conflicts of interest to disclose (Table 1).
3. Targeted/biological therapy

**Table 1:** Characteristics of included studies

**Study ID**	**Country**	**Study design**	***N*** ^a^	**Intervention**	**Treatment line**	**Co-interventions**	**Comparison**	**Conflicts of interest**
**Chemotherapy**								
Smeenk, 2005 [21]	Netherlands	OBS	83	5-FU	Not specified/Not clear	RT	BSC	NR
Takasawa, 2006 [22]	Japan	OBS	45	Gem	Not specified/Not clear	Endoscopic procedures	BSC	NR
Tada, 2008 [23]	Japan	OBS	167	Gem	Not specified/Not clear	RT	BSC	NR
Fujino, 2008 [24]	Japan	OBS	116	Gem	Not specified/Not clear	Palliative pancreatectomy	Not specified/Not clear	NR
Nakai, 2008 [25]	Japan	OBS	147	Gem	Not specified/Not clear	Stent	BSC	NR
Mukherjee, 2008 [26]	UK	OBS	294	Gem, Gem-based	Not specified/Not clear	RT	Not specified/Not clear	NR
Yamagishi, 2010 [27]	Japan	OBS	66	Gem	First line *Some patients also received second-line CT	-	BSC	NR
Matsumoto, 2011 [28]	Japan	OBS	68	Gem	Not specified/Not clear	-	BSC	NR
Hiramoto, 2011 [29]	Japan	OBS ^b^	128	Gem, S-1	Not specified/Not clear	-	BSC	NR
Hentic, 2011 [30]	France	OBS	38	Gem, Oxa	Not specified/Not clear	-	BSC	NR
Aldoss, 2011 [31]	USA	OBS	419	Not specified / not clear	Not specified/Not clear	-	Not specified/Not clear	Nothing to disclose
Vijayvergia, 2015 [32]	USA	OBS	579	5-FU, Gem, Iri, Platin, Taxanes	Not specified/Not clear	-	Not specified/Not clear	Nothing to disclose
Bednar, 2016 [33]^c^	USA	OBS	107	5-FU, Gem, Iri, Oxa, Pac	Not specified/Not clear	-	Not specified/Not clear	Declared^d^
Chakupurakal, 2017 [34]	Germany	OBS	324	5-FU, Cap, Gem, Oxa, nab-Pac, FOLFIRINOX, FOLFOX, FOLFIRI, Leucovorin	Not specified/Not clear	RT	BSC	Nothing to disclose
Henze, 2018 [35]	Germany	OBS ^b^	100	5-Fu, Gem, Iri, Oxa, Pac	Not specified/Not clear	RT	Not specified/Not clear	Nothing to disclose
Terashima, 2018 [36]	Japan	OBS	1085	Not specified / not clear	Not specified/Not clear	-	BSC	Nothing to disclose
Kang, 2020 [37]	South Korea	OBS	161	5-FU, Gem, Iri, Oxa, Leucovorin, Gem-based	Not specified/Not clear	Endoscopic procedures	BSC	Nothing to disclose
Iede, 2020 [38]	Japan	OBS	39	S-1	Second line	-	BSC	Nothing to disclose
Fukahori, 2020 [39]	Japan	OBS ^b^	255	Not specified / not clear	Not specified/Not clear	-	BSC	Declared^e^
Tralongo, 2020 [40]	USA	OBS ^b^	78	Not specified / not clear	Not specified/Not clear	-	BSC	Nothing to disclose
Andren Sandberg, 1983 [41]	Sweden	Q-Exp	47	5-FU, Vincristine, CCNU	First line	-	BSC	NR
Tsavaris, 1998 [42]	Greece	Q-Exp	90	5-FU, Epirubicin, Leucovorin	Not specified/Not clear	-	BSC	NR
Jiang, 2017 [43]	China	Q-Exp	47	S-1	First line	-	BSC	Nothing to disclose
The Gastrointestinal Tumor Study Group, 1979 [44]	USA	RCT	89	5-FU	First line	RT	Not specified/Not clear	Nothing to disclose
Mallinson, 1980 [45] ^c^	UK	RCT	40	5-FU, Cincristine, Cyclophsphamide, Methotrexate	First line	-	BSC	Nothing to disclose
Frey, 1981 [46]	USA	RCT	152	5-FU, CCNU	First line	Palliative surgery	Not specified/Not clear	Nothing to disclose
Palmer, 1994 [47]	UK	RCT	46	5-FU, Adriamycin, Mitomycin	Not specified/Not clear	-	Not specified/Not clear	Nothing to disclose
Glimelius, 1996 [48]	Sweden	RCT	53	5-FU, Leucovorin, Etoposide	First line	RT	BSC	Nothing to disclose
Takada, 1998 [49]	Japan	RCT	83	5-FU, Doxorubicin, Mitomycin	Not specified/Not clear	Palliative surgery	Not specified/Not clear	Nothing to disclose
Huguier, 2001 [50]	France	RCT	45	5-FU, Cisplatin, Leucovorin	First line	-	BSC	Nothing to disclose
Shinchi, 2002 [51]	Japan	RCT	31	5-FU	Not specified/Not clear	RT	Not specified/Not clear	Nothing to disclose
Ciuleanu, 2009 [52]	Romania	RCT	303	Glufosfamide	Second line	-	BSC	Declared^e^
Xinopoulos, 2008 [53] ^c^	Greece	RCT	49	Gem	First line	Stent	Not specified/Not clear	NR
Pelzer, 2011 [54] ^c^	Germany	RCT	46	5-FU, Oxa, Leucovorin	Second line	-	BSC	Nothing to disclose
Yip, 2006 [55]	Australia	SR	9 of 50	5-FU, Adriamycin, Cisplatin, Doxo, Mitomycin, Cyclophosphamide, Leucovirin, Vincristine, Etoposide, Metrothexate, CCNU, BCNU, FAM	Not specified/Not clear	RT, palliative surgery	BSC	NR
Chin, 2018 [56] ^c^	Australia	SR	6 of 60	5-FU, Cisplatin, Doxo, Gem, Mitomycin, CCNU, Vincristine, Leucovorin, Etoposide	First line	-	BSC	Nothing to disclose
Betge, 2018 [57]	Germany	Protocol for Q-Exp	-	Gem, Gem + nab-Pac	First line	-	BSC	Nothing to disclose
**Immunotherapy**								
Asahara, 2013 [58]	Japan	Q-Exp	112	HLA-A24-restricted peptide vaccine derived from KIF20A	Not specified/Not clear	-	BSC	Nothing to disclose
Jiang, 2017 [43]	China	Q-Exp	47	DC-CIK	First line	-	BSC	Nothing to disclose
Oortgiesen, 2010 [59]	USA	RCT	154	PAS	Not specified/Not clear	-	Placebo	Declared^f^
Gilliam, 2012 [60] ^c^	UK	RCT	154	G17DT: antigastrin immunogen	First line	-	Placebo	Nothing to disclose
**Targeted/biological therapy**								
Henze, 2018 [35]	Germany	OBS ^b^	100	Erlotinib	Not specified/Not clear	RT	Not specified/Not clear	Nothing to disclose
Reni, 2013 [61] ^c^	Italy	RCT	56	Sunitinib	Not specified/Not clear	-	Not specified/Not clear	Declared^g^
Propper, 2014 [62] ^c^	UK	RCT	207	Erlotinib	Not specified/Not clear	-	Placebo	Declared^e^
Golan, 2019 [63] ^c^	Israel	RCT	154	Olaparib	Not specified/Not clear	-	Placebo	Declared^e^

*OBS* observational Study; *Q-Exp* quasi-experimental Study; *RCT* randomised clinical trial; *SR* systematic review; *5-FU* fluorouracil; *Gem* gemcitabine; *Oxa* oxaliplatin; *Iri* irinotecan; *Pac* paclitaxel; *Cap* capecitabine; *Doxo* doxorubicin; *DC-CIK* dendritic cells and cytokine induced killer cells; *PAS* polyclonal antibody stimulator; *RT* radiotherapy; *BSC* best supportive care; *NR* not reported

^**a**^Number of included participants for primary studies and number of included studies relevant to our clinical question/total of included studies for systematic reviews

^b^Congress abstract

^c^See references for additional publications in online resource 2

^d^Two authors reported consulting or advisory roles with Healthcare Companies

^e^Authors are employees or have stocks on the Healthcare Company that funded the study

^f^Authors reported financial interests in the study product

^g^First author received consulting fees from the Pharmaceutical Manufacturing Company

Four studies published from 2013 to 2019 compared targeted or biological therapy to either placebo (*n* = 2) or to an intervention not clearly specified (*n* = 2) for advanced PC. Three were RCTs and one was an observational study and included a number of participants that ranged from 56 to 207.

Two studies evaluated erlotinib, one olaparib, and one sunitinib, all without specifying the line of therapy (Table 1).

All studies reported their conflicts of interest, of which one (25%) %) stated that there were no conflicts of interest to disclose (Table 1).

**Fig. 2:**
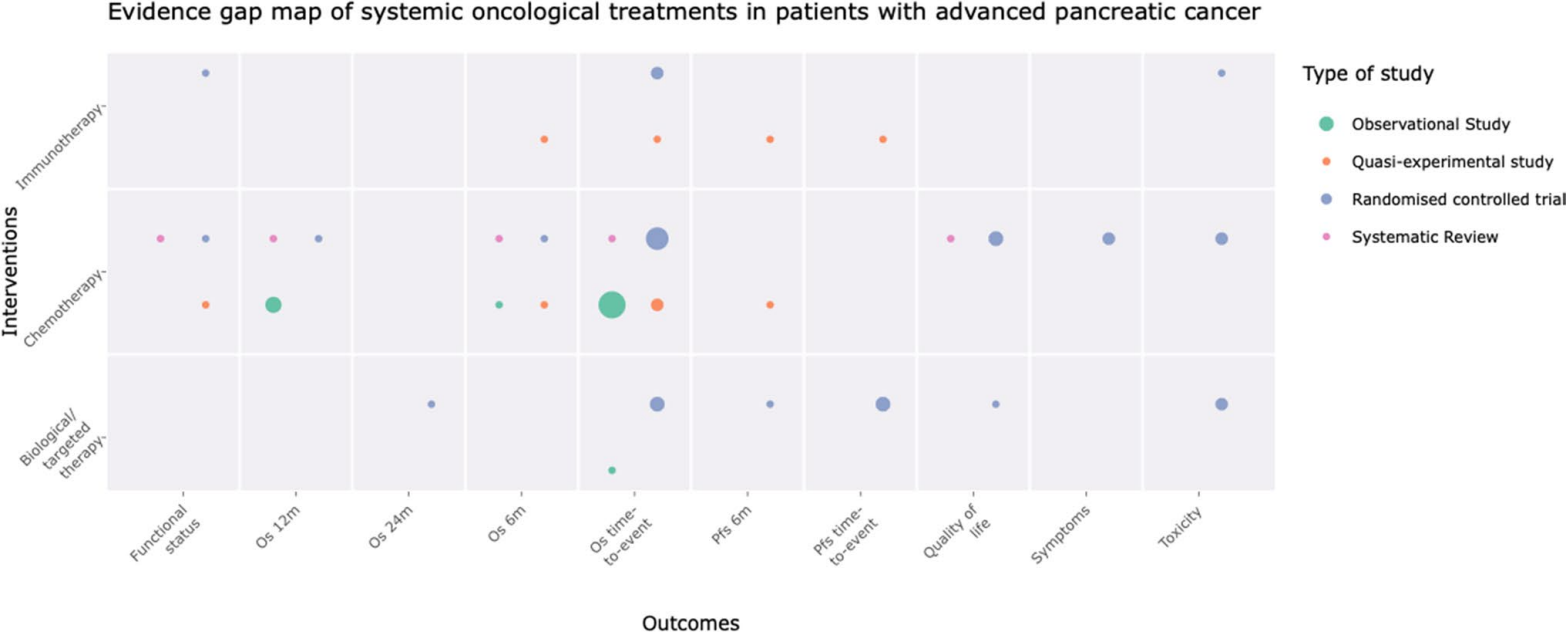
Evidence gap map of Systemic Oncological Treatments in patients with advanced pancreatic cancer

Figure 2 shows an overall summary of the evidence retrieved, classified by type of SOT administered, reported outcomes, and study design.

### Outcomes

**Fig. 3:**
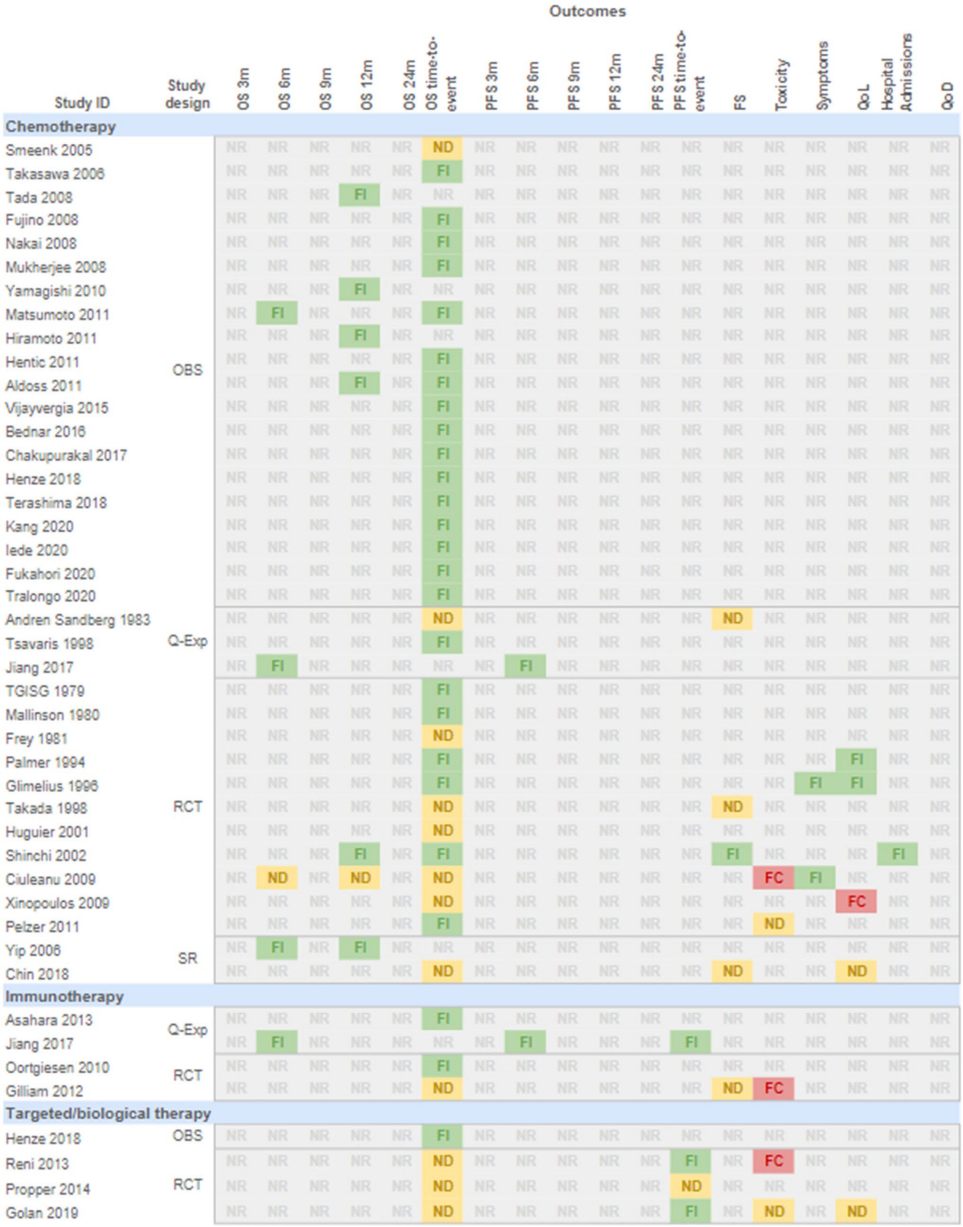
Direction of the effects reported by each study for each outcome. *OBS* observational study; *Q-Exp* quasi-experimental study; *RCT *randomised clinical trial; *SR* systematic review; *OS* overall survival; *PFS* progression-free survival; *m* months; *FS* functional status; *QoL* quality of life; *QoD* quality of death; *FI* favours intervention (Systemic Oncological Treatment); *ND* no differences; *FC* favours comparison (Best Supportive Care/placebo/not specified); *NR* not reported. *TGISG*  The Gastrointestinal Study Group

1. Chemotherapy
  All the 36 included studies reported survival-related outcomes, with most reporting an effect favouring the intervention [22–40, 42–45, 47, 48, 51, 54, 55]. Functional status was reported by four studies, including one SR [56], two RCTs [49, 51], one quasi-experimental design [41], with most showing no difference between both interventions, and only one RCT favouring the intervention [51]. Quality of life showed different results among the included studies, with a SR showing no difference [56], two RCTs favouring the intervention [47, 48], and one favouring the comparison [53]. Few RCTs reported toxicity [52, 54], symptoms [48, 52], and hospital admissions [51], and no study reported outcomes related to quality of death. Figure 3 provides a summary of the direction of the effect reported by each study for each outcome.
2. Immunotherapy
  All four included studies reported survival-related outcomes, with most reporting an effect favouring the intervention [43, 58, 59]. One RCT reported no differences between the interventions in functional status and results in favour of the control group for toxicity [59]. None of the studies reported symptoms related to the disease, quality of life, admissions to the hospital or quality of death (Fig. 3).
3. Targeted/biological therapy

Four studies reported overall survival, with three RCTs showing no difference [61–63] and one observational design favouring the intervention [35]. PFS was reported by the same three RCTs, with two favouring the intervention [61, 63] and one showing no difference [62]. Toxicity was reported in two RCTs, one favouring the control group [61], and one showing no difference [63]. Quality of life was reported by one RCT showing no differences between interventions [63]. None of the studies reported functional status, symptoms related to the disease, admissions to the hospital or quality of death (Fig. 3).

## Discussion

Our scoping review offers a broad overview of the currently available evidence of primary and secondary studies on the effectiveness and safety of SOTs compared to BSC, placebo or no SOT for patients with advanced PC. The evidence map presents results from 42 studies, 40 of which were either observational (*n* = 20) or experimental studies (*n* = 20), and with most of them reporting survival outcomes favouring the use of SOTs. Few studies reported other outcomes, with most reporting no difference in terms of functional status, most reporting results in favour of BSC in terms of toxicity, and heterogeneous results in terms of quality of life. Symptoms related to the disease and hospital admissions were scarcely reported, and no study reported outcomes related to quality of death.

Our exhaustive search resulted in the inclusion of only 42 studies to answer our question. Most studies evaluated chemotherapy as the intervention, and a high proportion were conducted before the year 2000. This could be due to newer studies tending to assess the comparative effectiveness of two SOTs, rather than with BSC as a sole comparator [64, 65]. Moreover, some authors may even consider it unethical to conduct an experimental study with one arm receiving only BSC, since most guidelines recommend SOTs as the standard of care [66]. However, we consider that the trade-off between survival and other relevant outcomes, such as toxicity and quality of life, is not clear enough and could justify the conduction of RCTs comparing active treatment against supportive care in patients with advanced PC at the EoL.

Almost all observational studies reported results favouring SOT in terms of survival. In contrast, about half of the RCTs assessing chemotherapy or immunotherapy and all RCTs assessing biological/targeted therapies reported no difference for the same outcome. This difference may be explained by the higher risk of selection bias in the observational studies, since it was frequent that the patients’ initial clinical assessment determined the type of therapy prescribed. Additionally, we do not know the degree of exhaustiveness of the disclosure of conflicts of interest since it was not reported by the authors of over 50% of the studies. Therefore, it is difficult to examine the extent to which potential conflicts may be associated with findings favouring SOTs.

Overall, there were very few studies reporting outcomes other than survival-related ones and none of the observational studies reported a non-survival outcome. Among the other methodological designs, toxicity was reported in only five studies, which can be explained by our inclusion of reports that assessed toxicity only as a comparison, that is, being reported in both the intervention and the control group. Some studies did provide data about toxicity in the intervention arm, but no information about the control, which limited the interpretation of the results. Lastly, we included only two SRs, which may be due to our strict eligibility criteria for this type of studies.

Our evidence map shows the current landscape of existing research and highlights evidence gaps. Our map shows that a small number of studies have been conducted to assess SOT compared to BSC, and even less reported critical patient centred outcomes. We found absolute evidence gaps, meaning no study reporting data, in the following outcomes: quality of death for all SOTs; quality of life for immunotherapy, and symptoms related to the disease, admissions to the hospital and quality of death for immunotherapy and biological/targeted therapies. In addition, it is important to note that caution is necessary when interpreting the study results that show a potential benefit of some treatments. The reporting of information about the lines of treatment is so scarce that patients with unequal prognosis would wrongfully be equated, which is something particularly relevant in a scenario where the more advanced the incurable disease the less likely it is that treatments will provide any benefit [67]. Therefore, it makes it very difficult to determine which specific patients are the ones that might benefit from specific lines of treatments and the limit beyond which there is no evidence to start a new therapeutic regimen [68]. In consequence, we claim for a more detailed description of administered treatments (i.e., number of cycles, previous treatments, co-interventions).

The main limitation of our review is related to the interpretation of the magnitude of effect of the results. Since this study was designed as a scoping review and evidence mapping, we planned to descriptively show only the direction of the effect of each SOT compared to BSC for the considered outcomes, as reported by the respective authors, but we did not consider reporting the magnitude of the effect nor to perform a quantitative summary of the results (e.g. meta-analysis). Also, we did not assess the risk of bias of each included study. However, our study has several strengths. To our knowledge, this is the only scoping review and evidence map of SOTs compared to BSC in patients with advanced PC. We conducted a comprehensive search including seven electronic databases, with a screening and data extraction process involving independent reviewers to minimise possible errors. Furthermore, we looked for all potentially relevant outcomes for patients at the EoL and we present the results in a reader-friendly graphical display, which strengthened the visibility of both primary and secondary research. In addition to this, we plan to conduct a new SR to update previous ones and include available RCTs to date. We will follow rigorous guidelines to assess the studies’ risk of bias and explore the magnitude of effect of the reported outcomes.

The use of SOTs will likely expand and become a common intervention for advanced PC. However, our results show that much about their effectiveness and safety, when compared to BSC, is still unknown. This uncertainty presents a challenge for health professionals, patients, and their relatives since it is necessary for clinicians to have objective criteria and relevant information in order to weigh potential benefits and side effects before prescribing treatments. Additionally, healthcare professionals must ensure that the patients understand this balance, especially in their particular context.

There is increasing recognition of the need to prioritise patient-centred communication and to have a focus on the patients’ goals of care [5]. For patients with advanced PC with a poor prognosis, these goals inevitably vary and may not necessarily be related only to an increase in survival [5]. Based on the results of our study, these other goals of care are not sufficiently reported. Therefore, it remains unclear if the potential benefits outweigh the risks of SOTs when compared to a conservative alternative such as BSC. In order to thoroughly assess the potential benefits of SOTs over BSC in advanced PC patients, future research should sufficiently report characteristics that will allow a better determination of the type of included patients based on their prognosis and previous treatments, and explicitly assess and report critical patient-centred outcomes such as toxicity, quality of life, admissions to the hospital and quality of death, through well powered, independent, and valid RCTs, included afterwards in the corresponding SR.

## Supplementary Information

Below is the link to the electronic supplementary material.

Supplementary file1 (DOCX 27 KB)

Supplementary file2 (DOCX 26 KB)

## Data Availability

Not applicable.

## References

[1] Sung H, Ferlay J, Siegel RL, Laversanne M, Soerjomataram I, Jemal A, Bray F (2021). Global Cancer Statistics 2020: GLOBOCAN Estimates of Incidence and Mortality Worldwide for 36 Cancers in 185 Countries. CA Cancer J Clin.

[2] National Cancer Institute. Surveillance Research Program, SEER*Explorer: An interactive website for SEER cancer statistics, US National Institutes of Health. Retreived April 6, 2022 from https://seer.cancer.gov/explorer/

[3] Hui D, Nooruddin Z, Didwaniya N, Dev R, De La Cruz M, Kim SH, Kwon JH, Hutchins R, Liem C, Bruera E (2014). Concepts and definitions for "actively dying," "end of life," "terminally ill," "terminal care," and "transition of care": a systematic review. J Pain Symptom Manage.

[4] PDQ Supportive and Palliative Care Editorial Board. (2022). Planning the Transition to End-of-Life Care in Advanced Cancer (PDQ®): Health Professional Version. In PDQ Cancer Information Summaries. National Cancer Institute (US). 26389513

[5] Mossman B, Perry LM, Walsh LE, Gerhart J, Malhotra S, Horswell R, Chu S, Raines AM, Lefante J, Blais CM, Miele L, Melancon B, Alonzi S, Voss H, Freestone L, Dunn A, Hoerger M (2021). Anxiety, depression, and end-of-life care utilization in adults with metastatic cancer. Psychooncology.

[6] Christenson ES, Jaffee E, Azad NS (2020). Current and emerging therapies for patients with advanced pancreatic ductal adenocarcinoma: a bright future. Lancet Oncol.

[7] Ducreux M, Cuhna AS, Caramella C, Hollebecque A, Burtin P, Goéré D, Seufferlein T, Haustermans K, Van Laethem JL, Conroy T, Arnold D, Guidelines Committee ESMO (2015). Cancer of the pancreas: ESMO Clinical Practice Guidelines for diagnosis, treatment and follow-up. Ann Oncol.

[8] Sohal D, Kennedy EB, Khorana A, Copur MS, Crane CH, Garrido-Laguna I, Krishnamurthi S, Moravek C, O'Reilly EM, Philip PA, Ramanathan RK, Ruggiero JT, Shah MA, Urba S, Uronis HE, Lau MW, Laheru D (2018). Metastatic Pancreatic Cancer: ASCO Clinical Practice Guideline Update. J Clin Oncol.

[9] Liu GF, Li GJ, Zhao H (2018). Efficacy and Toxicity of Different Chemotherapy Regimens in the Treatment of Advanced or Metastatic Pancreatic Cancer: A Network Meta-Analysis. J Cell Biochem.

[10] Hui D, De La Cruz M, Mori M, Parsons HA, Kwon JH, Torres-Vigil I, Kim SH, Dev R, Hutchins R, Liem C, Kang DH, Bruera E (2013). Concepts and definitions for "supportive care," "best supportive care," "palliative care," and "hospice care" in the published literature, dictionaries, and textbooks. Support Care Cancer.

[11] Zafar SY, Currow DC, Cherny N, Strasser F, Fowler R, Abernethy AP (2012). Consensus-based standards for best supportive care in clinical trials in advanced cancer. Lancet Oncol.

[12] Luthy C, Cedraschi C, Pugliesi A, Di Silvestro K, Mugnier-Konrad B, Rapiti E, Allaz AF (2011). Patients' views about causes and preferences for the management of cancer-related fatigue-a case for non-congruence with the physicians?. Support Care Cancer.

[13] Zafar SY, Alexander SC, Weinfurt KP, Schulman KA, Abernethy AP (2009). Decision making and quality of life in the treatment of cancer: a review. Support Care Cancer.

[14] Salazar J, Pérez-Bracchiglione J, Salas-Gama K, Antequera A, Auladell-Rispau A, Dorantes-Romandía R, Meade AG, Jesús Quintana M, Requeijo C, Rodríguez-Grijalva G, Santero M, Acosta-Dighero R, Solà I, Urrútia G, BonfillCosp X, Systemic Treatments for Advanced Digestive Cancer Research (2021). Efficacy of systemic oncological treatments in patients with advanced pancreatic cancer at high risk of dying in the short or medium-term: overview of systematic reviews. European J Cancer (Oxford, England : 1990).

[15] Bragge P, Clavisi O, Turner T, Tavender E, Collie A, Gruen RL (2011). The Global Evidence Mapping Initiative: scoping research in broad topic areas. BMC Med Res Methodol.

[16] Tricco AC, Lillie E, Zarin W, O'Brien KK, Colquhoun H, Levac D, Moher D, Peters M, Horsley T, Weeks L, Hempel S, Akl EA, Chang C, McGowan J, Stewart L, Hartling L, Aldcroft A, Wilson MG, Garritty C, Lewin S, Straus SE (2018). PRISMA Extension for Scoping Reviews (PRISMA-ScR): Checklist and Explanation. Ann Intern Med.

[17] Pérez-Bracchiglione J, Salazar J, Santero M, Requeijo C, Rodriguez Grijalva G, Acosta-Dighero R, Meza N, Salas Gama K, SelvaOlid A, Meade AG, Quintana Ge MJ, Urrutia Sola I, Bonfill X, Bottaro Parra D, Trujillo L, Cantero Fortiz Y 2022 Efficacy of systemic oncological treatments in patients with advanced, non-intestinal digestive cancer at high risk of dying in the middle and short term: Evidence synthesis Open Science Framework https://osf.io/7chx6/

[18] Methley AM, Campbell S, Chew-Graham C, McNally R, Cheraghi-Sohi S (2014). PICO, PICOS and SPIDER: a comparison study of specificity and sensitivity in three search tools for qualitative systematic reviews. BMC Health Serv Res.

[19] Krnic Martinic M, Pieper D, Glatt A, Puljak L (2019). Definition of a systematic review used in overviews of systematic reviews, meta-epidemiological studies and textbooks. BMC Med Res Methodol.

[20] Haddaway, N. R., & Keenan, C. (2021). evimappr: an R package for creating bubble plots for evidence maps. https://github.com/nealhaddaway/evimappr/

[21] Smeenk HG, de Castro SM, Jeekel JJ, Kazemier G, Busch OR, Incrocci L, Erdmann J, Hop WC, Gouma DJ, van Eijck CH (2005). Locally advanced pancreatic cancer treated with radiation and 5-fluorouracil: a first step to neoadjuvant treatment?. Dig Surg.

[22] Takasawa O, Fujita N, Kobayashi G, Noda Y, Ito K, Horaguchi J (2006). Endoscopic biliary drainage for patients with unresectable pancreatic cancer with obstructive jaundice who are to undergo gemcitabine chemotherapy. World J Gastroenterol.

[23] Tada M, Arizumi T, Nakai Y, Sasaki T, Kogure H, Togawa O, Matsubara S, Tsujino T, Hirano K, Sasahira N, Isayama H, Kawabe T, Omata M (2008). Efficacy of gemcitabine for locally advanced pancreatic cancer: comparison with 5-fluorouracil-based chemoradiotherapy. Chemotherapy.

[24] Fujino Y, Sakai T, Kuroda Y (2008). Palliative pancreatectomy with postoperative gemcitabine for patients with advanced pancreatic cancer. J Gastroenterol.

[25] Nakai Y, Isayama H, Kawabe T, Tsujino T, Yoshida H, Sasaki T, Tada M, Arizumi T, Yagioka H, Kogure H, Togawa O, Ito Y, Matsubara S, Hirano K, Sasahira N, Omata M (2008). Efficacy and safety of metallic stents in patients with unresectable pancreatic cancer receiving gemcitabine. Pancreas.

[26] Mukherjee S, Hudson E, Reza S, Thomas M, Crosby T, Maughan T (2008). Pancreatic cancer within a UK cancer network with special emphasis on locally advanced non-metastatic pancreatic cancer. Clin Oncol Royal College Radiol Great Britain.

[27] Yamagishi Y, Higuchi H, Izumiya M, Sakai G, Iizuka H, Nakamura S, Adachi M, Hozawa S, Takaishi H, Hibi T (2010). Gemcitabine as first-line chemotherapy in elderly patients with unresectable pancreatic carcinoma. J Gastroenterol.

[28] Matsumoto K, Miyake Y, Kato H, Kawamoto H, Imagawa A, Toyokawa T, Nakatsu M, Ando M, Hirohata M, Yamamoto K (2011). Effect of low-dose gemcitabine on unresectable pancreatic cancer in elderly patients. Digestion.

[29] Hiramoto S, Nishida Y, Hieda N, Mizuguchi A, Kakiuhi N, Yasumura S, Kuriyama K, Tanabe W, Hidaka K, Honjo H, Hasegawa K, Kondou M, Nishikawa K, Miyake N (2011). Retrospective study of chemotheraphy for unresectable advanced pancreatic cancer [Abstract]. Ann Oncol.

[30] Hentic O, Dreyer C, Rebours V, Zappa M, Lévy P, Raymond E, Ruszniewski P, Hammel P (2011). Gemcitabine in elderly patients with advanced pancreatic cancer. World J Gastroenterol.

[31] Aldoss IT, Tashi T, Gonsalves W, Kalaiah RK, Fang X, Silberstein P, Ganti AK, Subbiah S (2011). Role of chemotherapy in the very elderly patients with metastatic pancreatic cancer - A Veterans Affairs Cancer Registry analysis. J Geriatric Oncol.

[32] Vijayvergia N, Dotan E, Devarajan K, Hatahet K, Rahman F, Ricco J, Lewis B, Gupta S, Cohen SJ (2015). Patterns of care and outcomes of older versus younger patients with metastatic pancreatic cancer: A Fox Chase Cancer Center experience. J Geriatric Oncol.

[33] Bednar F, Ocuin LM, Steve J, Zenati MS, Winters S, Hogg ME, Bahary N, Zeh HJ, Zureikat AH (2016). FOLFIRINOX and gemcitabine/nab-paclitaxel efficacy in the treatment of locally advanced unresectable pancreatic adenocarcinoma [Abstract from 2016 ASCO Annual Meeting]. J Clin Oncol.

[34] Chakupurakal G, Feiten S, Burkhard O, Reiser M, Ehscheidt P, Weide R (2017). Successful Evidence-Based Treatment of Patients with Advanced Pancreatic Cancer in Community-Based Oncology Group Practices. Oncol Res Treatment.

[35] Henze L, Harder P, Kragl B, Murua Escobar H, Grosse-Thie C, Junghanss C (2018). Approved palliative chemotherapy regimens in unresectable pancreatic cancer: About 1/3 of patients in clinical routine show differences to trial entry criteria [Abstract]. Oncol Res Treatment.

[36] Terashima T, Yamashita T, Sakai A, Ohta H, Hinoue Y, Toya D, Kawai H, Yonejima M, Urabe T, Noda Y, Mizukoshi E, Kaneko S (2018). Treatment patterns and outcomes of unresectable pancreatic cancer patients in real-life practice: a region-wide analysis. Jpn J Clin Oncol.

[37] Kang J, Lee SH, Choi JH, Paik WH, Ahn DW, Jeong JB, Ryu JK, Kim YT (2020). Folfirinox chemotherapy prolongs stent patency in patients with malignant biliary obstruction due to unresectable pancreatic cancer. Hepatobiliary Pancreat Diseas Int: HBPD INT.

[38] Iede K, Yamada T, Kato R, Ueda M, Tsuda Y, Nakashima S, Ohta K, Matsuyama J, Ikenaga M, Tominaga S (2020). Efficacy of S-1 in second-line chemotherapy after nab-paclitaxel plus gemcitabine for patients with advanced pancreatic cancer. Cancer reports (Hoboken, NJ).

[39] Fukahori M, Okabe Y, Shimokawa M, Otsuka T, Koga F, Ueda Y, Nakazawa J, Komori A, Arima S, Makiyama A, Taguchi H, Honda T, Ushijima T, Miwa K, Shibuki T, Nio K, Ide Y, Ureshino N, Mitsugi K, Shirakawa T (2020). Efficacy of second-line chemotherapy after standard combination chemotherapy in patients with metastatic pancreatic cancer: The results from the NAPOLEON study [Abstract from 2020 Gastrointestinal Cancers Symposium]. J Clin Oncol.

[40] Tralongo AC, Sehovic M, Rodriquenz MG, Negrete Najar JP, Sam C, Extermann M (2020). Chemotherapy vs best supportive care in octogenarian and older stage IV pancreatic cancer patients [Abstract]. Ann oncol.

[41] Andrén-Sandberg A, Holmberg JT, Ihse I (1983). Treatment of unresectable pancreatic carcinoma with 5-fluorouracil, vincristine, and CCNU. Scand J Gastroenterol.

[42] Tsavaris N, Tentas K, Tzivras M, Kosmas C, Kalachanis N, Katsikas M, Dimitrakopoulos A, Papastratis G, Macheras A, Karatzas G, Sechas M (1998). Combined epirubicin, 5-fluorouracil and folinic acid vs no treatment for patients with advanced pancreatic cancer: a prospective comparative study. J Chemo (Florence, Italy).

[43] Jiang N, Qiao G, Wang X, Morse MA, Gwin WR, Zhou L, Song Y, Zhao Y, Chen F, Zhou X, Huang L, Hobeika A, Yi X, Xia X, Guan Y, Song J, Ren J, Lyerly HK (2017). Dendritic Cell/Cytokine-Induced Killer Cell Immunotherapy Combined with S-1 in Patients with Advanced Pancreatic Cancer: A Prospective Study. Clin Cancer Res.

[44] The Gastrointestinal Tumor Study Group (1979). A multi-institutional comparative trial of radiation therapy alone and in combination with 5-fluorouracil for locally unresectable pancreatic carcinoma The Gastrointestinal Tumor Study Group. Ann Surg.

[45] Mallinson CN, Rake MO, Cocking JB, Fox CA, Cwynarski MT, Diffey BL, Jackson GA, Hanley J, Wass VJ (1980). Chemotherapy in pancreatic cancer: results of a controlled, prospective, randomised, multicentre trial. BMJ.

[46] Frey C, Twomey P, Keehn R, Elliott D, Higgins G (1981). Randomized study of 5-FU and CCNU in pancreatic cancer: report of the Veterans Administration Surgical Adjuvant Cancer Chemotherapy Study Group. Cancer.

[47] Palmer KR, Kerr M, Knowles G, Cull A, Carter DC, Leonard RC (1994). Chemotherapy prolongs survival in inoperable pancreatic carcinoma. Br J Surg.

[48] Glimelius B, Hoffman K, Sjödén PO, Jacobsson G, Sellström H, Enander LK, Linné T, Svensson C (1996). Chemotherapy improves survival and quality of life in advanced pancreatic and biliary cancer. Ann Oncol.

[49] Takada T, Nimura Y, Katoh H, Nagakawa T, Nakayama T, Matsushiro T, Amano H, Wada K (1998). Prospective randomized trial of 5-fluorouracil, doxorubicin, and mitomycin C for non-resectable pancreatic and biliary carcinoma: multicenter randomized trial. Hepatogastroenterology.

[50] Huguier M, Barrier A, Valinas R, Flahault A, Adloff M, Pezet D, Jaeck D, Millat B, French University Association for Surgical Research (2001). Randomized trial of 5-fluorouracil, leucovorin and cisplatin in advanced pancreatic cancer. Hepato-gastroenterology.

[51] Shinchi H, Takao S, Noma H, Matsuo Y, Mataki Y, Mori S, Aikou T (2002). Length and quality of survival after external-beam radiotherapy with concurrent continuous 5-fluorouracil infusion for locally unresectable pancreatic cancer. Int J Radiat Oncol Biol Phys.

[52] Ciuleanu TE, Pavlovsky AV, Bodoky G, Garin AM, Langmuir VK, Kroll S, Tidmarsh GT (2009). A randomised Phase III trial of glufosfamide compared with best supportive care in metastatic pancreatic adenocarcinoma previously treated with gemcitabine. European journal of cancer (Oxford, England : 1990).

[53] Xinopoulos D, Dimitroulopoulos D, Karanikas I, Fotopoulou A, Oikonomou N, Korkolis D, Kouroumalis E, Antsaklis G, Vassilopoulos P, Paraskevas E (2008). Gemcitabine as palliative treatment in patients with unresectable pancreatic cancer previously treated with placement of a covered metal stent A randomized controlled trial. Journal of BUON.

[54] Pelzer U, Schwaner I, Stieler J, Adler M, Seraphin J, Dörken B, Riess H, Oettle H (2011). Best supportive care (BSC) versus oxaliplatin, folinic acid and 5-fluorouracil (OFF) plus BSC in patients for second-line advanced pancreatic cancer: a phase III-study from the German CONKO-study group. Eur J Cancer.

[55] Yip D, Karapetis C, Strickland A, Steer CB & Goldstein D (2006). Chemotherapy and radiotherapy for inoperable advanced pancreatic cancer. The Cochrane database of systematic reviews, (3), CD002093. 10.1002/14651858.CD002093.pub2 Update in: Cochrane Database Syst Rev. 2009 (4):CD002093.

[56] Chin V, Nagrial A, Sjoquist K, O'Connor CA, Chantrill L, Biankin AV, Scholten RJ, Yip D (2018). Chemotherapy and radiotherapy for advanced pancreatic cancer. The Cochrane Database Syst Rev.

[57] Betge J, Chi-Kern J, Schulte N, Belle S, Gutting T, Burgermeister E, Jesenofsky R, Maenz M, Wedding U, Ebert M, Haertel N (2018). A multicenter phase 4 geriatric assessment directed trial to evaluate gemcitabine +/− nab-paclitaxel in elderly pancreatic cancer patients (GrantPax). BMC Cancer.

[58] Asahara S, Takeda K, Yamao K, Maguchi H, Yamaue H (2013). Phase I/II clinical trial using HLA-A24-restricted peptide vaccine derived from KIF20A for patients with advanced pancreatic cancer. J Transl Med.

[59] Oortgiesen JM, DiMichele LA, Weidman JR, Gunto VT, Soeder T, Cato A, Sutton L (2010). Immune response to gastrin-17 and survival in gastrointestinal cancers [Abstract from 2010 ASCO Annual Meeting]. J Clin Oncol.

[60] Gilliam AD, Broome P, Topuzov EG, Garin AM, Pulay I, Humphreys J, Whitehead A, Takhar A, Rowlands BJ, Beckingham IJ (2012). An international multicenter randomized controlled trial of G17DT in patients with pancreatic cancer. Pancreas.

[61] Reni M, Cereda S, Milella M, Novarino A, Passardi A, Mambrini A, Di Lucca G, Aprile G, Belli C, Danova M, Bergamo F, Franceschi E, Fugazza C, Ceraulo D, Villa E (2013). Maintenance sunitinib or observation in metastatic pancreatic adenocarcinoma: a phase II randomised trial. Eur J Cancer.

[62] Propper D, Davidenko I, Bridgewater J, Kupcinskas L, Fittipaldo A, Hillenbach C, Klughammer B, Ducreux M (2014). Phase II, randomized, biomarker identification trial (MARK) for erlotinib in patients with advanced pancreatic carcinoma. Ann Oncol.

[63] Golan T, Hammel P, Reni M, Van Cutsem E, Macarulla T, Hall MJ, Park JO, Hochhauser D, Arnold D, Oh DY, Reinacher-Schick A, Tortora G, Algül H, O'Reilly EM, McGuinness D, Cui KY, Schlienger K, Locker GY, Kindler HL (2019). Maintenance Olaparib for Germline BRCA-Mutated Metastatic Pancreatic Cancer. N Engl J Med.

[64] Singh RR, O'Reilly EM (2020). New Treatment Strategies for Metastatic Pancreatic Ductal Adenocarcinoma. Drugs.

[65] Katayama ES, Hue JJ, Bajor DL, Ocuin LM, Ammori JB, Hardacre JM, Winter JM (2020). A comprehensive analysis of clinical trials in pancreatic cancer what is coming down the pike?. Oncotarget.

[66] Castro M (2007). Placebo versus best-available-therapy control group in clinical trials for pharmacologic therapies: which is better?. Proc Am Thorac Soc.

[67] Kordes M, Yu J, Malgerud O, GustafssonLiljefors M, Löhr J (2019). Survival Benefits of Chemotherapy for Patients with Advanced Pancreatic Cancer in A Clinical Real-World Cohort. Cancers.

[68] Hua J, Shi S, Liang D, Liang C, Meng Q, Zhang B, Ni Q, Xu J, Yu X (2018). Current status and dilemma of second-line treatment in advanced pancreatic cancer: is there a silver lining?. Onco Targets Ther.

