# Supplementary material for: Systemic oncological treatments in patients with advanced pancreatic cancer: a scoping review and evidence map
Source: Support Care Cancer. 2023 Jan 9;31(2):100. doi: 10.1007/s00520-022-07564-8 (PMC9829581; doi:10.1007/s00520-022-07564-8)
Supplement: Supplementary file 1 — Supplementary file1 (DOCX 27 KB) [file 520_2022_7564_MOESM1_ESM.docx]

**Systemic oncological treatments in patients with advanced pancreatic cancer: A scoping review and evidence map.**

**Supportive Care in Cancer**

Salazar J (1), Bracchiglione J (1,2), Acosta-Dighero R (3), Meza N (2), Meade A (1), Quintana MJ (1,6), Requeijo C (1), Rodríguez-Grijalva G (1), Santero M (1), Selva A (4, 6), Solà I (1,5,6), Bonfill X (1,5,6), Appropriateness of Systemic Oncological Treatments for Advanced Cancer (ASTAC) Research Group.

(1) Iberoamerican Cochrane Centre, Biomedical Research Institute Sant Pau (IIB Sant Pau), Barcelona, Spain

(2) Interdisciplinary Centre for Health Studies (CIESAL), Universidad de Valparaíso, Viña del Mar, Chile

(3) Department of Physical Therapy, Faculty of Medicine, University of Chile, Santiago, Chile

(4) Corporació Sanitària Parc Taulí, Sabadell, Spain

(5) CIBER Epidemiología y Salud Pública (CIBERESP), Barcelona, Spain

(6) Universitat Autònoma Barcelona, Barcelona, Spain.

Corresponding author: Xavier Bonfill Cosp, C/ Sant Antoni Maria Claret, 167, Pavelló 18, planta 0. 08025. Barcelona, España. Teléfono: +34 93 553 78 14 Fax: +34 93 553 78 09. [xbonfill@santpau.cat](mailto:xbonfill@santpau.cat)

**SEARCH STRATEGY FOR PUBMED**

| #1 | ("Gastrointestinal Neoplasms"[Mesh:NoExp] OR "Esophageal Neoplasms"[Mesh] OR "Stomach Neoplasms"[Mesh] OR "Liver Neoplasms"[Mesh] OR "Biliary Tract Neoplasms"[Mesh] OR "Pancreatic Neoplasms"[Mesh]) |
| --- | --- |
| #2 | ((esophag*[Title] OR oesophag*[Title] OR stomach*[Title] OR gastric*[Title] OR gastroesophag*[Title] OR liver*[Title] OR hepatic*[Title] OR hepatocel*[Title] OR biliary tract*[Title] OR bile duct*[Title] OR gallbladder*[Title] OR gall bladder*[Title] OR pancreas*[Title] OR pancreatic*[Title] OR gastrointestinal*[Title]) AND (cancer*[Title] OR carcinom*[Title] OR neoplasm*[Title] OR tumor*[Title] OR tumour*[Title] OR malignan*[Title] OR adenocar*[Title] OR oncolog*[Title])) |
| #3 | (#1 OR #2) |
| #4 | ("Palliative Care"[Mesh] OR "Terminal Care"[Mesh] OR "Neoplasm Metastasis"[Mesh]) |
| #5 | (palliative*[Title/Abstract] OR end of life*[Title/Abstract] OR end of live*[Title/Abstract] OR terminal*[Title/Abstract] OR metasta*[Title/Abstract] OR BSC[Title/Abstract] OR supportive care*[Title/Abstract] OR advanced*[Title/Abstract] OR unresect*[Title/Abstract] OR irresect*[Title/Abstract] OR nonresect*[Title/Abstract] OR non resect*[Title/Abstract] OR inopera*[Title/Abstract] OR unopera*[Title/Abstract] OR nonopera*[Title/Abstract] OR non opera*[Title/Abstract] OR non-opera*[Title/Abstract] OR stage IV[Title/Abstract]) |
| #6 | (#4 OR #5) |
| #7 | (#3 AND #6) |
| #8 | ("Antineoplastic Protocols"[Mesh] OR "Chemoradiotherapy"[Mesh] OR "Induction Chemotherapy"[Mesh] OR "Maintenance Chemotherapy"[Mesh] OR "Consolidation Chemotherapy"[Mesh]) |
| #9 | (antineoplastic*[Title/Abstract] OR antineoplasic*[Title/Abstract] OR chemotherap*[Title/Abstract] OR chemoradiotherap*[Title/Abstract] OR radiochemotherap*[Title/Abstract] OR carboplatin*[Title/Abstract] OR cisplatin*[Title/Abstract] OR fluorouracil*[Title/Abstract] OR 5-FU[Title/Abstract] OR capecitabine*[Title/Abstract] OR docetaxel*[Title/Abstract] OR epirucibin*[Title/Abstract] OR irinotecan*[Title/Abstract] OR oxaliplatin*[Title/Abstract] OR paclitaxel*[Title/Abstract] OR trifluridine*[Title/Abstract] OR tipiracil*[Title/Abstract]) |
| #10 | (#8 OR #9) |
| #11 | ("Molecular Targeted Therapy"[Mesh] OR "Antibodies, Monoclonal"[Mesh] OR "Cancer Vaccines"[Mesh]) |
| #12 | (Target*[Title/Abstract] OR antibod*[Title/Abstract] OR immunotherap*[Title/Abstract] OR vaccine[Title/Abstract] OR vaccines[Title/Abstract] OR vaccination[Title/Abstract] OR HER2[Title/Abstract] OR HER-2[Title/Abstract] OR egfr[Title/Abstract] OR VEGF*[Title/Abstract] OR HGF[Title/Abstract] OR MET[Title/Abstract] OR claudin*[Title/Abstract] OR MMP  9[Title/Abstract][JP1] OR tyrosine kinase inhibit*[Title/Abstract] OR trastuzumab[Title/Abstract] OR bevacizumab[Title/Abstract] OR rilotumumab[Title/Abstract] OR onartuzumab[Title/Abstract] OR ramucirumab[Title/Abstract] OR cetuximab[Title/Abstract] OR panitumumab[Title/Abstract] OR nimotuzumab[Title/Abstract] OR claudiximab[Title/Abstract] OR apatinib[Title/Abstract] OR lapatinib[Title/Abstract] OR regorafenib[Title/Abstract] OR everolimus[Title/Abstract] OR nivolumab[Title/Abstract] OR pembrolizumab[Title/Abstract] OR avelumab[Title/Abstract] OR durvalumab[Title/Abstract] OR ipilimumab[Title/Abstract] OR checkpoint inhibit*[Title/Abstract]) |
| #13 | (#11 OR #12) |
| #14 | (#10 OR #13) |
| #15 | (#7 AND #14) |
| #16 | (animals [mh] NOT humans [mh]) |
| #17 | #15 NOT #16 |
