# Supplementary material for: Systemic oncological treatments in patients with advanced pancreatic cancer: a scoping review and evidence map
Source: Support Care Cancer. 2023 Jan 9;31(2):100. doi: 10.1007/s00520-022-07564-8 (PMC9829581; doi:10.1007/s00520-022-07564-8)
Supplement: Supplementary file 2 — Supplementary file2 (DOCX 26 KB) [file 520_2022_7564_MOESM2_ESM.docx]

**Systemic oncological treatments in patients with advanced pancreatic cancer: A scoping review and evidence map.**

**Supportive Care in Cancer**

Salazar J (1), Bracchiglione J (1,2), Acosta-Dighero R (3), Meza N (2), Meade A (1), Quintana MJ (1,6), Requeijo C (1), Rodríguez-Grijalva G (1), Santero M (1), Selva A (4, 6), Solà I (1,5,6), Bonfill X (1,5,6), Appropriateness of Systemic Oncological Treatments for Advanced Cancer (ASTAC) Research Group.

(1) Iberoamerican Cochrane Centre, Biomedical Research Institute Sant Pau (IIB Sant Pau), Barcelona, Spain

(2) Interdisciplinary Centre for Health Studies (CIESAL), Universidad de Valparaíso, Viña del Mar, Chile

(3) Department of Physical Therapy, Faculty of Medicine, University of Chile, Santiago, Chile

(4) Corporació Sanitària Parc Taulí, Sabadell, Spain

(5) CIBER Epidemiología y Salud Pública (CIBERESP), Barcelona, Spain

(6) Universitat Autònoma Barcelona, Barcelona, Spain.

Corresponding author: Xavier Bonfill Cosp, C/ Sant Antoni Maria Claret, 167, Pavelló 18, planta 0. 08025. Barcelona, España. Teléfono: +34 93 553 78 14 Fax: +34 93 553 78 09. [xbonfill@santpau.cat](mailto:xbonfill@santpau.cat)

**References of additional publications of the same study**

| Study ID | Reference | Additional References |
| --- | --- | --- |
| Bednar, 2016 | Bednar, F., Ocuin, L. M., Steve, J., Zenati, M.S., Winters, S., Hogg, M. E., Bahary, N., Zeh, H. J., & Zureikat, A. H. (2016) FOLFIRINOX and gemcitabine/nab-paclitaxel efficacy in the treatment of locally advanced unresectable pancreatic adenocarcinoma [Abstract from 2016 ASCO Annual Meeting]. Journal of Clinical Oncology, 34(4_suppl), 399–399. http://dx.doi.org/10.1200/jco.2016.34.4_suppl.399 | - **(Abstract)** Bednar, F., Ocuin, L. M., Steve, J., Zenati, M.S., Winters, S., Hogg, M. E., Bahary, N., Zeh, H. J., Lii, H., & Zureikat, A. H. (2016) Folfirinox and gemcitabine/nab-paclitaxel demonstrate improved survival in locally advanced unresectable pancreatic adenocarcinoma [Abstract from 69th Cancer Symposium of the Society of Surgical Oncology]. Annals of Surgical Oncology, 23(1_suppl), S175–S176. |
| Mallinson, 1980 | Mallinson, C. N., Rake, M. O., Cocking, J. B., Fox, C. A., Cwynarski, M. T., Diffey, B. L., Jackson, G. A., Hanley, J., & Wass, V. J. (1980). Chemotherapy in pancreatic cancer: results of a controlled, prospective, randomised, multicentre trial. British medical journal, 281(6255), 1589–1591. https://doi.org/10.1136/bmj.281.6255.1589 | - **(Abstract)** Mallinson, C. N., Rake, M.O., & Fox, C.A. (1977). Final results of a controlled trial of chemotherapy for inoperable pancreatic cancer [Abstract]. Irish Journal of Medical Science, 146(suppl.1) |
| Xinopoulos, 2008 | Xinopoulos, D., Dimitroulopoulos, D., Karanikas, I., Fotopoulou, A., Oikonomou, N., Korkolis, D., Kouroumalis, E., Antsaklis, G., Vassilopoulos, P., & Paraskevas, E. (2008). Gemcitabine as palliative treatment in patients with unresectable pancreatic cancer previously treated with placement of a covered metal stent. A randomized controlled trial. Journal of B.U.ON. : official journal of the Balkan Union of Oncology, 13(3), 341–347. | - Xinopoulos, D., Dimitroulopoulos, D., Fotopoulou, A., Korkolis, D., Tsamakidis, K., Kypreos, D., Basioukas, S., Pasavela, S., Loukou, A. & Paraskevas, E. (2009). Palliation with Previously Gemcitabine in Patients with Advanced Pancreatic Cancer Treated with the Placement of a Covered Metal Biliary Stent. Annals of Gastroenetology, 22(1), 0–15. |
| Pelzer, 2011 | Pelzer, U., Schwaner, I., Stieler, J., Adler, M., Seraphin, J., Dörken, B., Riess, H., & Oettle, H. (2011). Best supportive care (BSC) versus oxaliplatin, folinic acid and 5-fluorouracil (OFF) plus BSC in patients for second-line advanced pancreatic cancer: a phase III-study from the German CONKO-study group. European journal of cancer (Oxford, England : 1990), 47(11), 1676–1681. https://doi.org/10.1016/j.ejca.2011.04.011 | - **(Trial Resgistry)** Nr. ISRCTN52780546 - **(Abstract)** Oettle, H., Pelzer, U., Stieler, J., Hilbig, A., Roll, L., Schwaner, I., Adler, M., Detken, S., Dorken, B., & Riess, H. (2005). Oxaliplatin/folinic acid/5-fluorouracil [24h] (OFF) plus best supportive care versus best supportive care alone (BSC) in second-line therapy of gemcitabine-refractory advanced pancreatic cancer (CONKO 003) [Abstract from 2005 ASCO Annual Meeting]. Journal of Clinical Oncology, 23(16_suppl), 4031–4031. <https://doi.org/10.1200/jco.2005.23.16_suppl.4031> |
| Chin, 2018 | Chin, V., Nagrial, A., Sjoquist, K., O'Connor, C. A., Chantrill, L., Biankin, A. V., Scholten, R. J., & Yip, D. (2018). Chemotherapy and radiotherapy for advanced pancreatic cancer. The Cochrane database of systematic reviews, 3(3), CD011044. https://doi.org/10.1002/14651858.CD011044.pub2 | - **(Protocol)** Nagrial, A., Chantrill, L., Chin, V., Sjoquist, K., O'Connor, C. A., & Yip, D. (2013) Pharmacologic and radiotherapeutic interventions for advanced pancreatic cancer. Cochrane Database of Systematic Reviews. Art. No.: CD010584. https://doi.org//10.1002/14651858.CD010584 |
| Gilliam, 2012 | Gilliam, A. D., Broome, P., Topuzov, E. G., Garin, A. M., Pulay, I., Humphreys, J., Whitehead, A., Takhar, A., Rowlands, B. J., & Beckingham, I. J. (2012). An international multicenter randomized controlled trial of G17DT in patients with pancreatic cancer. Pancreas, 41(3), 374–379. https://doi.org/10.1097/MPA.0b013e31822ade7e | - **(Abstract)** Gilliam, A. D., Topuzov, E. G., Garin, A. M., Pulay, I., Broome, P., Watson, S. A., Rowlands, B. J., Takhar, A., & Beckingham, I. J. (2004). Randomised, double blind, placebo-controlled, multi-centre, group-sequential trial of G17DT for patients with advanced pancreatic cancer unsuitable or unwilling to take chemotherapy [Abstract from 2004 ASCO Annual Meeting]. Journal of Clinical Oncology, 22(14_suppl), 2511–2511. https://doi.org/10.1200/jco.2004.22.90140.2511 |
| Reni, 2013 | Reni, M., Cereda, S., Milella, M., Novarino, A., Passardi, A., Mambrini, A., Di Lucca, G., Aprile, G., Belli, C., Danova, M., Bergamo, F., Franceschi, E., Fugazza, C., Ceraulo, D., & Villa, E. (2013). Maintenance sunitinib or observation in metastatic pancreatic adenocarcinoma: a phase II randomised trial. European journal of cancer (Oxford, England : 1990), 49(17), 3609–3615. https://doi.org/10.1016/j.ejca.2013.06.041 | - **(Abstract)** Reni, M., Cereda, S., Milella, M., Novarino, A., Passardi, A., Mambrini, A., Di Lucca, G., Ferrari, L., Belli, C., Danova, M., Bergamo, F., Franceschi, E., Rovati, B., Fugazza, C., Ceraulo, D., & Villa, E. (2012). Maintenance sunitinib (MS) or observation (O) in metastatic pancreatic adenocarcinoma (MPA): Clinical and translational results of a phase II randomized trial (NCT00967603) [Abstract from 2012 ASCO Annual Meeting]. Journal of Clinical Oncology, 30(15_suppl), 4017–4017. https://doi.org/10.1200/jco.2012.30.15_suppl.4017 |
| Propper, 2014 | Propper, D., Davidenko, I., Bridgewater, J., Kupcinskas, L., Fittipaldo, A., Hillenbach, C., Klughammer, B., & Ducreux, M. (2014). Phase II, randomized, biomarker identification trial (MARK) for erlotinib in patients with advanced pancreatic carcinoma. Annals of oncology : official journal of the European Society for Medical Oncology, 25(7), 1384–1390. https://doi.org/10.1093/annonc/mdu176 | - **(Abstract)** Ducreux, M., Davidenko, I., Bridgewater, J., Kupcinskas, L., Johannsdottir, H., Van Der Horst, T., Klughammer, B., & Propper, D. (2011). Invstigatins potential biomarkers for survival with erlotinib in patients with advanced pancreatic cancer - Results of the phase II BO21129 study [Abstract]. European Journal of Cancer, 47 (1_suppl), S464–S464 |
| Golan, 2019 | Golan, T., Hammel, P., Reni, M., Van Cutsem, E., Macarulla, T., Hall, M. J., Park, J. O., Hochhauser, D., Arnold, D., Oh, D. Y., Reinacher-Schick, A., Tortora, G., Algül, H., O'Reilly, E. M., McGuinness, D., Cui, K. Y., Schlienger, K., Locker, G. Y., & Kindler, H. L. (2019). Maintenance Olaparib for Germline BRCA-Mutated Metastatic Pancreatic Cancer. The New England journal of medicine, 381(4), 317–327. https://doi.org/10.1056/NEJMoa1903387 | - **(Abstract)** Hochhauser, D., Kindler, H., Hammel, P., Reni, M., Van Cutsem, E., Macarulla, T., Hall, M. J., Park, J. O., Arnold, D., Oh, D. -Y., Reinacher-Schick, A., Tortora, G., Algül, H., O'Reilly, E. M., McGuinness, D., Cui, K., Schlienger, K., Locker, G., & Golan, T. (2020). Assessing clinical benefit of olaparib maintenance treatment in subgroups of patients with germline BRCA mutation (gBRCAm) and metastatic pancreatic cancer: Phase III POLO trial [Abstract]. Annals of Oncology, 31(4_suppl), S940–S941. <https://doi.org/10.1016/j.annonc.2020.08.2010> - **(Abstract)** Hall, M. J., Golan, T., Hammel, P., Reni, M., Van Cutsem, E., Macarulla, T., Park, J. O., Hochhauser, D., Arnold, D., Oh, D. -Y., Reinacher-Schick, A. C., Tortora, G., Algül, H., O'Reilly, E. M., McGuinness, D., Cui, K., Joo, S., Yoo, H.K., Patel, N., & Kindler, H. L. (2020). Pancreatic cancer (PaC)-specific health-related quality of life (HRQoL) with maintenance olaparib (O) in patients (pts) with metastatic (m) PaC and a germline BRCA mutation (gBRCAm): Phase III POLO trial [Abstract from 2020 Gastrointestinal Cancers Symposium]. Journal of Clinical Ongology, 38(4_suppl), 648–648. <https://doi.org/10.1200/JCO.2020.38.4_suppl.648> - **(Abstract)** Schwartz, L. H., Kindler, H. L., Hammel, P., Reni, M., Van Cutsem, E., Macarulla, T., Hall, M.J., Oh Park, J., Hochhauser, D., Arnold, D., Oh, D. -Y., Reinacher-Schick, A., Tortora, G., Alguel, H., O'Reilly, E. M., Fromageau, J., Ghiorghiu, D. C., McGuinness, D., Locker, G. Y., & Golan, T. (2020). POLO: Radiologic assessment of the impact of maintenance olaparib in patients (pts) with metastatic pancreatic cancer (mPaC) [Abstract from 2020 ASCO Annual Meeting]. Journal of Clinical Oncology, 38(15_suppl). <https://doi.org/10.1200/JCO.2020.38.15_suppl.e16800> - **(Abstract)** Yoo, H. K., Kindler, H. L., McCutcheon, S., McGuinness, D., Patel, N., Hettle, R., Goodbody, R., Joo, S., Locker, G. Y., & Golan, T. (2020). POLO: Quality-adjusted (QA) progression-free survival (PFS) and patient (pt)-centered outcomes with maintenance olaparib in pts with metastatic pancreatic cancer (mPaC) [Abstract from 2020 ASCO Annual Meeting]. Journal of Clinical Oncology, 38(15_suppl), 4626–4626. <https://doi.org/10.1200/JCO.2020.38.15_suppl.4626> - **(Abstract)** Hammel, P., Kindler, H. L., Reni, M., Van Cutsem, E., Macarulla Mercade, T., Hall, M. J., Park, J. O., Hochhauser, D., Arnold, D., Oh, D. -Y., Reinacher-Schick, A., Tortora, G., Algül, H., O'Reilly, E. M., McGuinness, D., Cui, K. Y., Joo, S., Yoo, H. K., Patel, N., & Golan, T. (2019). POLO: Health-related quality of life (HRQoL) of olaparib maintenance treatment versus placebo in patients with a germline BRCA mutation and metastatic pancreatic cancer (mPC) [Abstract]. Annals of Oncology, 30(5_suppl), V254–V255. <https://doi.org/10.1093/annonc/mdz422.004> - Hammel, P., Kindler, H. L., Reni, M., Van Cutsem, E., Macarulla, T., Hall, M. J., Park, J. O., Hochhauser, D., Arnold, D., Oh, D. Y., Reinacher-Schick, A., Tortora, G., Algül, H., O'Reilly, E. M., McGuinness, D., Cui, K. Y., Joo, S., Yoo, H. K., Patel, N., Golan, T., … POLO Investigators (2019). Health-related quality of life in patients with a germline BRCA mutation and metastatic pancreatic cancer receiving maintenance olaparib. Annals of oncology : official journal of the European Society for Medical Oncology, 30(12), 1959–1968. https://doi.org/10.1093/annonc/mdz406 - Golan, - T., Kindler, H. L., Park, J. O., Reni, M., Macarulla, T., Hammel, P., Van Cutsem, E., Arnold, D., Hochhauser, D., McGuinness, D., Locker, G. Y., Goranova, T., Schatz, P., Liu, Y. Z., & Hall, M. J. (2020). Geographic and Ethnic Heterogeneity of Germline BRCA1 or BRCA2 Mutation Prevalence Among Patients With Metastatic Pancreatic Cancer Screened for Entry Into the POLO Trial. Journal of clinical oncology : official journal of the American Society of Clinical Oncology, 38(13), 1442–1454. https://doi.org/10.1200/JCO.19.01890 |
